# ICP-MS As a Contributing Tool to Nontarget Screening (NTS) Analysis for Environmental Monitoring

**DOI:** 10.1021/acs.est.4c00504

**Published:** 2024-07-10

**Authors:** Jörg Feldmann, Helle Rüsz Hansen, Thomas Molnár Karlsson, Jan H. Christensen

**Affiliations:** † TESLA-Analytical Chemistry, Institute of Chemistry, University of Graz, Universitätsplatz 1, Graz 8010, Austria; ‡ Danish Environmental Protection Agency, Tolderlundsvej 5, Odense C 5000, Denmark; § Analytical Chemistry group, Department of Plant and Environmental Sciences, University of Copenhagen, Frederiksberg C 1871 , Denmark

**Keywords:** quantification without standards, environmental monitoring, heteroatoms, exposome, mass balance, mass spectrometry, liquid chromatography, unknown pollutants, chemicals of emerging concern

## Abstract

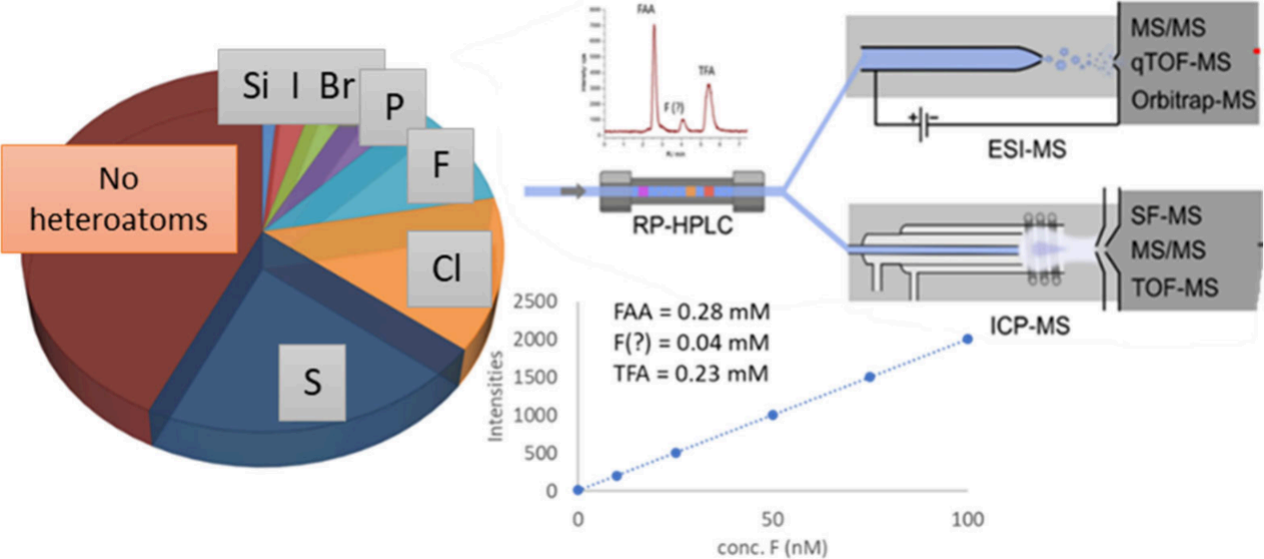

Due to the increasing number of chemicals released into the environment, nontarget screening (NTS) analysis is a necessary tool for providing comprehensive chemical analysis of environmental pollutants. However, NTS workflows encounter challenges in detecting both known and unknown pollutants with common chromatography high-resolution mass spectrometry (HRMS) methods. Identification of unknowns is hindered by limited elemental composition information, and quantification without identical reference standards is prone to errors. To address these issues, we propose the use of inductively coupled plasma mass spectrometry (ICP-MS) as an element-specific detector. ICP-MS can enhance the confidence of compound identification and improve quantification in NTS due to its element-specific response and unambiguous chemical composition information. Additionally, mass balance calculations for individual elements (F, Br, Cl, etc.) enable assessment of total recovery of those elements and evaluation of NTS workflows. Despite its benefits, implementing ICP-MS in NTS analysis and environmental regulation requires overcoming certain shortcomings and challenges, which are discussed herein.

## Abstract 2

ICP-MS, when used as a detector for LC or GC, can enhance confidence in compound identification and aid in quantification of compounds in environmental monitoring for which no standards exist.

## 1 Introduction: Beyond Targeted Approaches in Environmental Monitoring

Monitoring of environmental pollution relies heavily on targeted methods focusing on selected analytes described in regulatory directives. For example, monitoring the chemical status of water bodies in the EU is based on highly targeted quantitative analytical methods fulfilling the criteria defined in the COMMISSION DIRECTIVE 2009/90/EC of 31 July 2009.

In these methods, chemical standards are used for identification and quantification using calibration solutions. This enables compound identification based on mass spectrum and retention time and provides reliable concentration estimates, often by correcting for matrix effects and analyte response using isotopically labeled internal standards. Proficiency tests and round robins are organized (e.g., FAPAS and others) to guarantee high quality quantitative data and accreditation according to ISO/IEC 17025. Accredited laboratories provide transparent data, enabling regulators to make informed administrative decisions. Maximum concentration levels are typically discussed only when analytes can be quantified with high confidence, as seen with inorganic arsenic in rice.^1^

Relying solely on targeted approaches in monitoring programs omits a vast number of known and unknown pollutants, their transformation products (TPs), as well as replacement products of banned organic chemicals.^2^ The continuously evolving list of monitored compounds highlights the increasing number of chemicals released into the environment, only a fraction of which is fully understood in terms of their toxicity and environmental fate.^3,4^ For example, perfluorooctanoic acid (PFOA) and perfluorooctanesulfonate (PFOS), banned by the Stockholm Convention, have been substituted by other, less studied substances like GenX.^5^ These replacements may constitute environmental threats due to their persistence, mobility, and toxicity.

With increasing awareness of the importance of suspected and unknown compounds, there has been growing interest in methods aimed at analyzing a wider range of compounds and chemical groups. These are collectively known as suspect screening and NTS methods, depending on the analytical and data science workflow that is employed. NTS is rapidly gaining traction in the field of environmental monitoring, which can be seen in the dramatic increase in the number of publications since 2013 (Figure S1). Additionally, untargeted analysis is used in metabolomics, while chemical fingerprinting finds applications in characterizing complex mixtures in the petrochemical industry.^6^

Despite the interest in developing NTS workflows, several issues persist, which limit their application in environmental monitoring, particularly related to the uncertainty of confidence in compound identification,^7^ quantification,^8^ and loss of compounds in the analytical workflows. To overcome these challenges, we argue that ICP-MS can bring valuable contributions to the NTS toolbox by supporting:

(1) identification of chemicals of emerging concern (CECs),
(2) quantification of compounds for which no standards exist,
(3) detection of hidden compounds, which cannot be determined in the analytical workflow

## 2 Workflows for Measuring the Unknowns and NTS

Suspect screening and NTS workflows entail sample preparation, separation and detection, followed by data processing of the raw data to detect, identify, and quantify compounds. Aqueous samples often undergo solid-phase extraction (SPE) with generic sorbents such as hydrophilic–lipophilic balance (HLB) materials. Analytical separation includes reversed phase liquid chromatography (RP-LC), and gas chromatography (GC). To broaden the analytical platforms, other separation techniques such as hydrophilic lipophilic interaction chromatography (HILIC),^16^ ion chromatography (IC)^9,10^ and supercritical fluid chromatography (SFC)^11,33^ have also been employed to include the more polar compounds. Additional techniques like ion mobility and two-dimensional chromatography enhance separation and improve annotation, and identification of CECs.^12−14^ Detection relies on high-resolution mass spectrometry (HRMS) for both suspect screening and NTS.

Suspect screening and NTS differ in data processing workflows for detecting, identifying, and quantifying chromatography peaks. Both suspect screening and NTS involve a series of preprocessing steps like peak detection, filtering, and retention time and signal intensity correction using software such as MS-Dial, patRoon or MZmine. Afterward, peak annotation, compound identification, prioritization, and concentration estimation are conducted. NTS operates with limited prior information about compounds, while suspect screening uses a predefined library of target searches via software like FindPFAS, based on retention index and mass spectral data. Unlike suspect screening, NTS avoids preselection enhancing its sensitivity to unknown and unsuspected compounds in the sample.

## 3 Current Challenges with NTS Approaches

### 3.1 Limited Information for Identification of Unknown Compounds

Annotation and identification of chromatographic features (*t*_r_ = retention time, *m*/*z*), and components (sets of *t*_r_, *m*/*z* features) rely heavily on compound structure and physio-chemical properties. Identification confidence levels follows frameworks such as the one developed by Schymanski and co-workers,^7^ which considers accurate mass and mass fragmentation spectra (see Figure S3). Furthermore, retention indexes can be used to increase the level of confidence.^15^ Unambiguous identification (level 1) requires confirmation using a reference standard, while level 2 relies on comparison of MS and MS^2^ information in external libraries. Additional approaches include assigning identification points based on retention index, accurate mass, mass fragments and isotope patterns, which can be done retrospectively.^11,16−18^ One of the major bottlenecks is the establishment of a molecular formulas (level 4). The chemical composition of an unknown compound can be deduced from the accurate mass, mass spectral fragmentation pattern, and isotope pattern, but for many compounds this remains challenging unless the highest mass resolution instruments are used (>100,000). Here the unambiguous detection of heteroelements, such as Cl, F, S, or P, can contribute to establish the molecular formula by limiting possible compositions within a certain mass window.

### 3.2 Quantification Uncertainty

Quantification poses another significant challenge in suspect screening and NTS due to limited availability of analytical standards and isotopically labeled internal standards for only a few compounds.^19^ It is therefore necessary to turn to tools for concentration estimation, which is often done using surrogate compounds to predict the response factors of suspect screening and NTS compounds.^20^ These surrogates share similarities with identified compounds in terms of retention time, since the matrix effecting the ionization is similar, structural features, or ionizability.^19,21,22^ Prediction methods for relative response factors using extensive reference standards training sets exist. While these methods offer reasonable concentration estimates for known compounds,^21^ interlaboratory studies have revealed significant variability in quantifying individual compounds without molecular identical standards. This variability can span more than 2 orders of magnitude, even when compounds with similar retention time and similar estimated ionizability were used.^8^ Despite advancements, such as including all ions in electrospray ionization as well as reliable ways to transfer between different instruments and chromatography methods,^22^ a significant need persists for additional tools to reliably quantify suspects and unknowns.

The uncertainty surrounding quantitative results limits the regulatory applicability of NTS. While suspect screening and NTS data aid in identifying relevant pollutants for targeted analysis, achieving accurate quantitative results poses challenges. Current methods often fail to meet the precision requirements specified in regulatory directives.^8^ Leveraging LC-ICP-MS for reliable element quantification could enhance NTS workflows, ensuring compliance with regulatory standards.

### 3.3 Loss of Analytes in Chemical Workflows

Despite aiming to include a wide range of compounds, preselections is evitable in suspect screening and NTS workflows due to varying recovery rates across compound groups (see e.g.,^9^). Although generic SPE sorbents are utilized, methods are always tailored toward certain compound classes with similar physio-chemical properties, thereby excluding others. Solutions to this challenge include employing techniques such as large-volume injection or online SPE to minimize sample preparation, and utilizing complementary chromatography methods (e.g., HILIC, SFC, and RP) to expand compound coverage and reduce biases. However, this approach may result in lower enrichment factors, higher detection limits, and increased matrix effects in the absence of cleanup. Ultimately, inherent bias leads to a reduction in the number of detectable compounds. For instance, very polar compounds can be overlooked in NTS of aqueous samples, due to insufficiently retention in RP-SPE and RP-LC workflows,^9,23^ while many nonpolar compounds may remain undetected due to low ionizability with electrospray ionization (ESI) or atmospheric pressure chemical ionization (APCI).^24^ Alternative chromatographic methods such as HILIC, SFC,^11^ and GC × GC, coupled with HRMS detection, can enhance the detection, identification, and quantification of compounds. However, they also add complexity to NTS workflows.

Taking into account all these factors, even NTS can detect only a limited number of compounds, and the degree of hidden compounds is difficult to quantify (e.g., not ionized PFAS^24^ or not in database in suspected screening^25^). Employing additional complementary methods can indeed increase the number of detectable compounds. However, it is important to note that one NTS workflow may only detect a fraction of the compounds present in a sample.

Despite its designation as nontargeted, NTS analysis unavoidably incorporates “targeted” elements due to decisions made in sample preparation and analytical workflows, influencing the compounds that can be detected. This makes all NTS inherently selective.^26^ Consequently, it is crucial to consider the potential loss of compound groups to identify and mitigate systematic biases in environmental monitoring.

## 4 Can ICP-MS Strengthen the NTS Toolbox?

### 4.1 Use of ICP-MS in Environmental Analysis

Ionization in ICP-MS occurs within the argon plasma, a hard ionization source that breaks down molecules into their constituent atoms and efficiently ionizes most elements in the periodic table. It has been used as an element-specific detector for more than 30 years, primarily utilized for the analysis of metals and semimetals, which ionize very efficiently due to their low ionization potential. However, all elements except O and N, due to their high background, can be analyzed with trace (ppb or μM) or ultratrace (ppt or nM) level detection limits. Methods for quantifying even notoriously challenging elements like F have been developed for ICP-MS analysis.^24,27,28^

The potential applications of ICP-MS are highlighted by the extensive list of compounds in the NORMAN Substance Database (SusDat), exceeding 100 000 entries. SusDat compiles suspect screening lists for environmental contaminants developed by the NORMAN network members.^29^ Upon closer examination, it is evident that 56% of these compounds contain a heteroatom detectable by ICP-MS. These elements are found in major CEC groups, such as F in PFAS, pesticides and pharmaceuticals, Br in flame retardants, and F and Cl in pesticides and pharmaceuticals (Figure S2).

So far, however, ICP-MS, has rarely been used for NTS analysis of CECs. However, the authors assert that integrating ICP-MS into the NTS tool-box can yield several benefits

(i) Enhancing confidence in identifying CECs.
(ii) Lowering quantification uncertainty for compounds for which no standards exist.
(iii) Detecting hidden compounds, which cannot be determined in the analytical workflow used.

**Figure 1:**
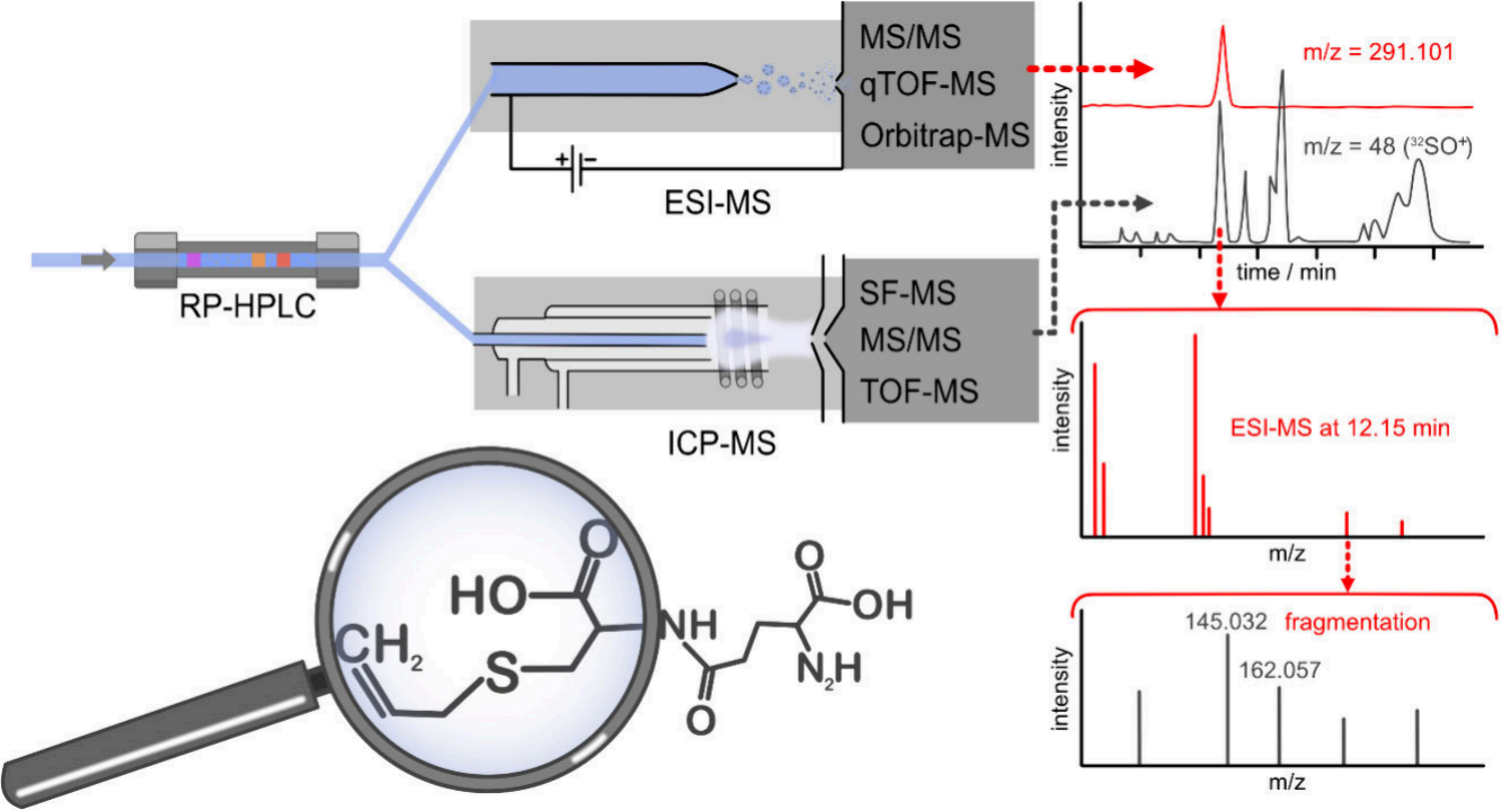
LC-ICP-MS/ESI-MS setup and workflow. Detection, identification and quantification of unknown compounds containing a heteroatom (here S).

This will help overcome current obstacles for integrating suspect screening and NTS as part of environmental monitoring programs. Particularly promising is the use of LC-ICP-MS in combination with LC-ESI-HRMS. This setup combines element-specific detection from ICP with molecular information from ESI, typically used in NTS. In this setup (see Figure 1), a valve is introduced after the LC to split the LC-flow between the ICP-MS and the ESI-MS system. The retention time of known analytes can subsequently be used to correct for differences in dead volumes between the two systems and align the data. This system simultaneously generates both element-specific and molecular information. Hansen and co-workers^30^ pioneered this approach using low resolution ESI-MS, later advancing to HRMS.^31^

### 4.2 Adding Information to Support Identification of CECs

LC-ICP-MS can contribute to the detection, identification, and quantification of unknown analytes. Compounds undetected in ESI-MS data due to coelution interferences or ion suppression matrix effects may be detected by the element-specific ICP-MS detection in this dual detection approach. This is particularly useful when the mass defect is insignificant, as seen with fluorinated pharmaceuticals and pesticides compared to perfluorinated carboxylic acids. It has been demonstrated recently for the identification of a transformation product of a PFAS (telomer alcohol 6:2) using *MZmine* for peak detection and identification. The more than 1000 detected features in *MZmine* could be reduced by fluorine detection with parallel ICP-MS to 21 potential TPs. This condensed list facilitated screening, leading to the identification of a novel TP of a known PFAS compound.^25^ This demonstrates the potential to augment the system for compound identification, introducing an additional level in the Schymanski scheme (see Figure S3) based on the unambiguous assignment of chemical composition, which is gained from the element-specific signal from ICP-MS matching the compound detection in ESI-HRMS. While partly achievable with ESI, especially for elements with characteristic isotope patterns, such as Br and Cl, ICP makes it possible to extend this approach to include all elements that can be analyzed by ICP-MS, including monoisotope elements such as As and F. This additional information can be integrated into the Identification Point System to extend the scoring system and help avoid false positives.^11^

### 4.3 Quantification without a Molecularly Identical Standard

The hard ionization of an argon plasma in ICP-MS provides an element-specific response, independent of chemical structure. Consequently, the response factor for compounds containing a heteroelement remains constant within a given matrix under isocratic elution. In gradient elution, the carbon enhancement effect occur due to changes in organic solvent content, but this can be overcome by post column infusion of an internal standard (e.g., a different element or isotope) to measure the matrix effect as a function of retention time.^32^ No other matrix effects are known, which makes ICP-MS an ideal detector to run in parallel with ESI-MS for quantification in NTS, requiring only an elemental standard as an internal standard to determine the response factor.

Several element-specific detection systems, including neutron activation, X-ray absorption, and graphite furnace atomic absorption, exhibit element-specific rather than molecular-specific responses. However, only ICP with atomic emission or mass spectrometry detection can easily be coupled to a variety of chromatography methods (e.g., GC, LC, and SFC) due to the possibility of continuous infusion into the ICP. Other detectors such as Atomic Emission Detection (AED), Nitrogen Phosphorus Detection (NPD), or Electron Capture Detection (ECD) also provide element-specific or element-selective detection with continuous infusion but are restricted to GC.

**Figure 2:**
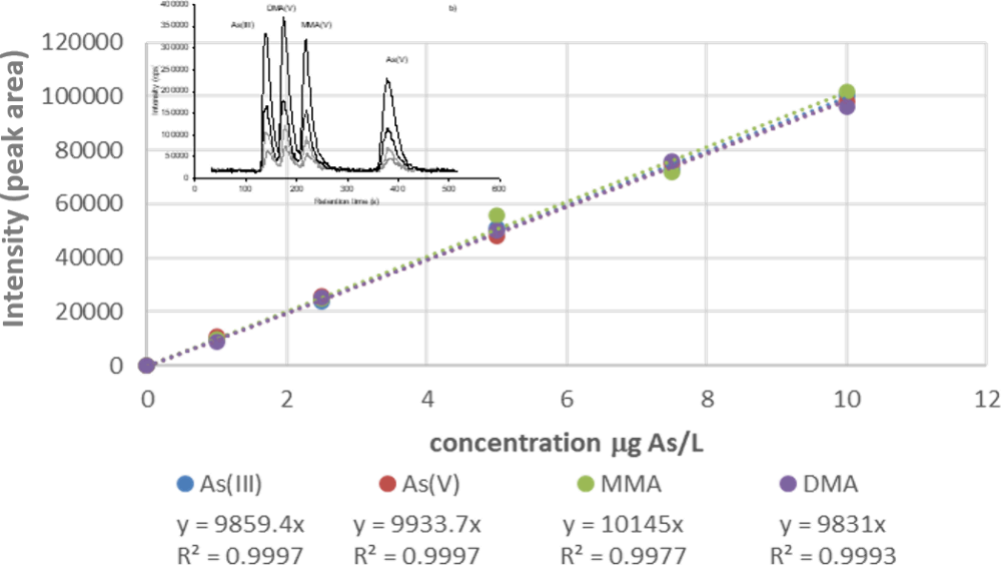
Four arsenic compounds (arsenite (As(III)), arsenate (As(V)), monomethylarsonic acid (MMA), dimethylarsinic acid (DMA)) are separated by LC-ICP-MS demonstrating compound independent quantification using LC-ICP-MS.

The compound independent calibration can be seen in Figure 2 for four As containing compounds. Similar calibrations can also be done for nonmetals such as F-containing compounds for PFAS analysis.^24,27^ Workflows incorporating ICP-MS can therefore be a strong contributor to reliable quantification in suspect screening and NTS, particular in scenarios where chemical structure is unknown or identical standards are unavailable. Hence, ICP-MS increases confidence in compound quantification by utilizing surrogate standards containing the same heteroatom (see Figure S4). This is particularly relevant for providing quantitative NTS data that can be used in regulatory contexts demanding high accuracy and precision. Additionally, it supports results obtained from other model prediction tools in LC-ESI-MS.^21,33^

### 4.4 Mass Balance for Elemental Analysis to Support Analytical Workflows

**Figure 3:**
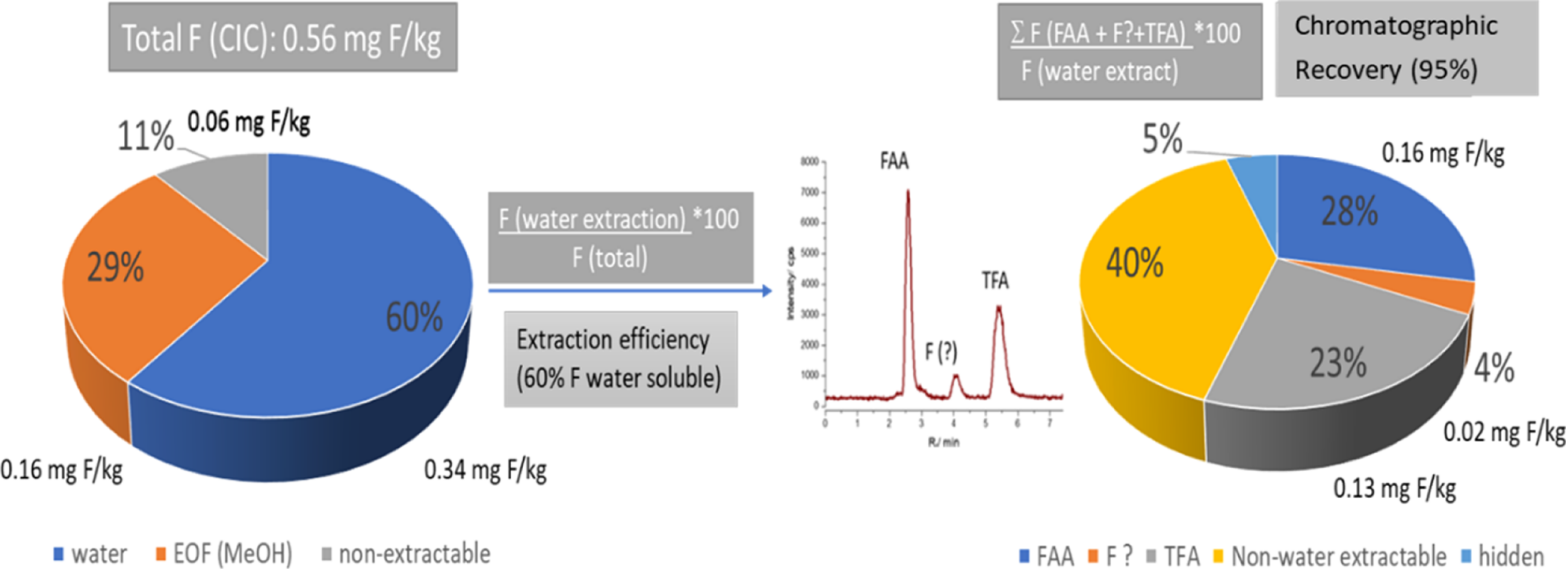
Mass balance approach in NTS of compounds containing one heteroelement, here F. The left pie-chart shows the loss during extraction of compounds, while the right pie-chart shows the distribution of fluorine-containing compounds including the unknown compound in the water extract.

ICP-MS enables the quantification of total element concentrations, facilitating the development and validation of workflows. With simple mass balance equations, losses during different steps can be quantified, which gives an indication of workflow performance.^26^ By determining the amount of an element extracted and left behind in the residue, the extraction efficiency of the element can be calculated. This makes it possible to quantify recovery of each analytical step (Figure 3). In the analysis step, an element-specific chromatogram can be obtained from the ICP-MS, enabling the quantification of individual compounds. The ratio of the sum of individual compounds to the total element concentration injected on the column yields chromatographic recovery. Total element concentrations, often obtained directly by ICP-MS analysis of the extract with or without prior digestion, help quantify compounds containing the element that do not elute or are below the detection limit, thus identifying hidden compounds.

To illustrate this further, consider an example of F-containing compounds in a solid environmental sample, as shown in Figure 3. The total fluorine concentration can be determined after full sample digestion followed by ICP-MS analysis or directly as solid in combustion ion chromatography (CIC). Fractionation reveals 11% was not extractable in water and methanol. Water-soluble highly polar fluorine compounds account for 60%, while organofluorines soluble in methanol make up 29% of fluorine (often called extractable organofluorine (EOF)). This allows for investigation of sample preparation, enabling quantification of nonextractable or hidden compounds. Inspection of the fluorine-specific chromatogram, reveals two compounds identified by retention time comparison with standards, confirmed by parallel use of ESI-HRMS. Additionally, an unknown fluorine-containing compound (labeled “F?”) was quantified using a fluorine-containing surrogate due to the compound-independent response in ICP-MS. The difference between the sum of all chromatographically identified fluorine-containing compounds and the total fluorine content in the water fraction gives the amounts of hidden compounds (here 5%). The ratio is used in speciation analysis and expressed as the chromatographic recovery (95%).

The ability to quantify the analytical recovery of elements enables more extensive method development and validation. This includes not only information about detected compounds but also the sum of compounds which are hidden. For example, total fluorine measurements in the extract as EOF are often compared to the sum of all quantified PFAS from LC-MS/MS or LC-HRMS. These investigations often reveal a large proportion of unidentified or hidden F-containing compounds in water samples, such as wastewater.^34^ Using this approach, it becomes evident whether individual PFAS are eluting from the column or if the EOF contains fluorine-containing polymers or nanoparticles that would not elute. This approach can also quantify F-containing compounds which do not ionize with ESI-HRMS.

## 5 Challenges and Future Developments for Element Analysis in NTS

### 5.1 Detection of Challenging Compounds by ICP-MS

A number of challenges exist that limit the usefulness of ICP-MS in NTS and environmental monitoring, indicating areas for future research.

Notably, ICP-MS struggles to detect nonmetal elements, particularly C, N, O, and halogens. While quantification challenges persist for elements like C, N and O due to high background concentrations, analytical performance can still be improved for many elements. ICP-HRMS has been used in the past for elements such as P and S which suffer from poly atomic interferences. Today, ICP-MS/MS have utilized reaction and collision cells to provide interference-free detection of the elements Cl, Br, S, and P. However, improvements are needed for F detection, despite advancements with methods based on the formation of BaF^+^ adducts.^24,27,28^ Detection limits for elements with high ionization potential such as F remain in the μg/L range as measured solution, which constitutes a challenge for ICP-MS and LC-ICP-MS in NTS workflows.

One way to improve element detection is through improved MS detection. The most used mass analyzer for ICP-MS is a single quadrupole. The advantages of a quadrupole system include its low cost, ability to perform both qualitative and quantitative analyses, and increased sensitivity by the use of selected ion monitoring mode. The major drawback of quadrupole analyzers for the use of ICP-MS in NTS workflows is that their scanning nature limits the acquisition rate and thereby provides poor detection limits for all-ion detection. An alternative mass analyzer for ICP-MS is ICP-TOF-MS (time-of-flight MS). It is a nonscanning mass analyzer that can be used for all elements and isotopes at the same time, which is advantageous for NTS. Additionally, it enables accurate measurement of isotope ratios of heteroelements of the transient signals coming from the LC and GC,^35^ enhancing compound identification confidence.

### 5.2 Decreased Sensitivity with Organic Solvents

Another challenge in LC-ICP-MS arises from the use of organic solvents, which requires the ICP instrument to be run in so-called “organic mode” where addition of oxygen gas is used to avoid carbon deposits and carrier gas flow is lowered to avoid plasma overload.^36^ However, this compromises sensitivity and long-term stability that hinders widespread LC-ICP-MS implementation.

Several approaches can address this challenge. One strategy involves using alternative organic solvents that are more compatible with ICP, either because their low vapor pressure means that smaller amounts enter the plasma, or higher eluent strength which means that lower percentage of the organic solvent is required. For instance, 1,2-hexanediol has been demonstrated as a viable solvent for LC-ICP-MS rather than methanol or acetonitrile typically used for RP-LC.^37^ Using SFC is another possible solution, since it gives elution conditions that are more amenable for the ionization in ICP-MS.^38^

### 5.3 Co-eluting Compounds

As ICP-MS detects individual elements, it is not possible to distinguish between coeluting compounds with the same heteroatom. Thus, identification or quantification biases can occur if two or more coeluting compounds contain the same heteroelement, either by wrongly assigning elemental composition or overestimating the ICP-MS signal from individual compounds. This presents a challenge, particularly for complex samples often analyzed with NTS. However, it is less likely that compounds with a measured heteroatom coelute, since fewer compounds exist than ionized organic molecules in a water sample. If coelution exists, then quantification of the coeluting compounds can only be given as a sum rather than reporting individual values.

The combined data from ICP-MS and ESI-MS can solve the issue by identifying individual features that can afterward be deconvoluted in the ICP-MS data.^32^ However, for compounds that are very poorly separated on the LC such as stereoisomers, or cases where coeluting compounds are not detectable by ESI-MS, this might not be possible. Another approach is to improve the LC separation using two-dimensional LC, which greatly improves the ability to separate compounds by using orthogonal retention mechanisms.^39^

### 5.4 Alternative Instruments for Element Analysis

In addition to ICP-MS, several other element detectors also exist that deserve attention. These include ICP coupled with atomic emission spectroscopy (AES), atomic emission detector with a helium plasma (AED), ECD, and NPD, all offering sensitive element detection. However, ICP-AES is often less sensitive and incapable of detecting different isotopes. NPD’s advantage lies in its ability to analyze N, which is not possible with ICP-MS. Nevertheless, its limited number of detectable elements and restrictions to GC coupling hinder widespread implementation. The same applies to an ECD, which is particularly useful for halogens. The ECD gives only electron affinity detection and cannot discriminate between halogens.

In summary, we advocate for increased focus on implementation of ICP-MS in suspect screening and NTS for environmental monitoring. The use of ICP-MS and LC-ICP-MS workflows shows promise in detecting and identifying unknown compounds, as well as improving quantification of suspect or unknown compounds where no reference standards exist. Additionally, compounds that are hidden with GC-MS and LC-HRMS analysis become quantifiable using ICP-MS. Thus, ICP-MS in NTS may provide a more comprehensive understanding of the identity and quantity of environmental pollutants, offering significant benefits for regulatory authorities.

## Supporting Information Available

Figure S1: Web of Science bibliographic search, sum of publications with topic matching “non-target screening”, “suspect screening” or “non-target analysis” between 1991 and 2023; FigureS2: Percentage of compounds containing heteroatoms that can be selectively detected by ICP-MS (from the NORMAN list of emerging contaminants); Figure S3: Identification Level scheme modified from Schymanski et al. 2014 by introducing ICP-MS information to the scheme as level 4b. S4: Confidence level scheme for quantification using NTS. (PDF)
