# Supplementary material for: ICP-MS As a Contributing Tool to Nontarget Screening (NTS) Analysis for Environmental Monitoring
Source: Environ Sci Technol. 2024 Jul 10;58(29):12755–62. doi: 10.1021/acs.est.4c00504 (PMC11271004; doi:10.1021/acs.est.4c00504)

Supplementary material:

ICP-MS as a contributing tool to non-target screening (NTS) analysis for environmental monitoring

Jörg Feldmann[1,], Helle Rüsz Hansen[2,*], Thomas Molnár Karlsson[3,*], Jan H. Christensen[3,*]*

[1]TESLA-Analytical Chemistry, Institute of Chemistry, University of Graz, Universitätsplatz 1, 8010 Graz, Austria

[2]Danish Environmental Protection Agency, Tolderlundsvej 5, 5000, Odense C, Denmark

[3]Department of Plant and Environmental Sciences, University of Copenhagen, 1871 Frederiksberg C, Denmark

*Corresponding authors: joerg.feldmann@uni-graz.at, herha@mst.dk, tmmk@plen.ku.dk, jch@plen.ku.dk

Table of content:

Figure S1: *Web of Science bibliographic search, sum of publications with topic matching "non-target screening", "suspect screening" or "non-target analysis" between 1991-2023, February 2024.*

Figure S2: Percentage of compounds containing heteroatoms which can be selectively detected by ICP-MS (from the NORMAN list of Emerging contaminants current status 106).

Figure S3: Identification Level scheme modified from Schymanski et al. 2014 by introducing ICP-MS information to the scheme as level 4b.

Figure S4: Confidence level scheme for quantification using NTS.

Figure S1:. *Web of Science bibliographic search, sum of publications with topic matching "non-target screening", "suspect screening" or "non-target analysis" between 1991-2023, February 2024.*

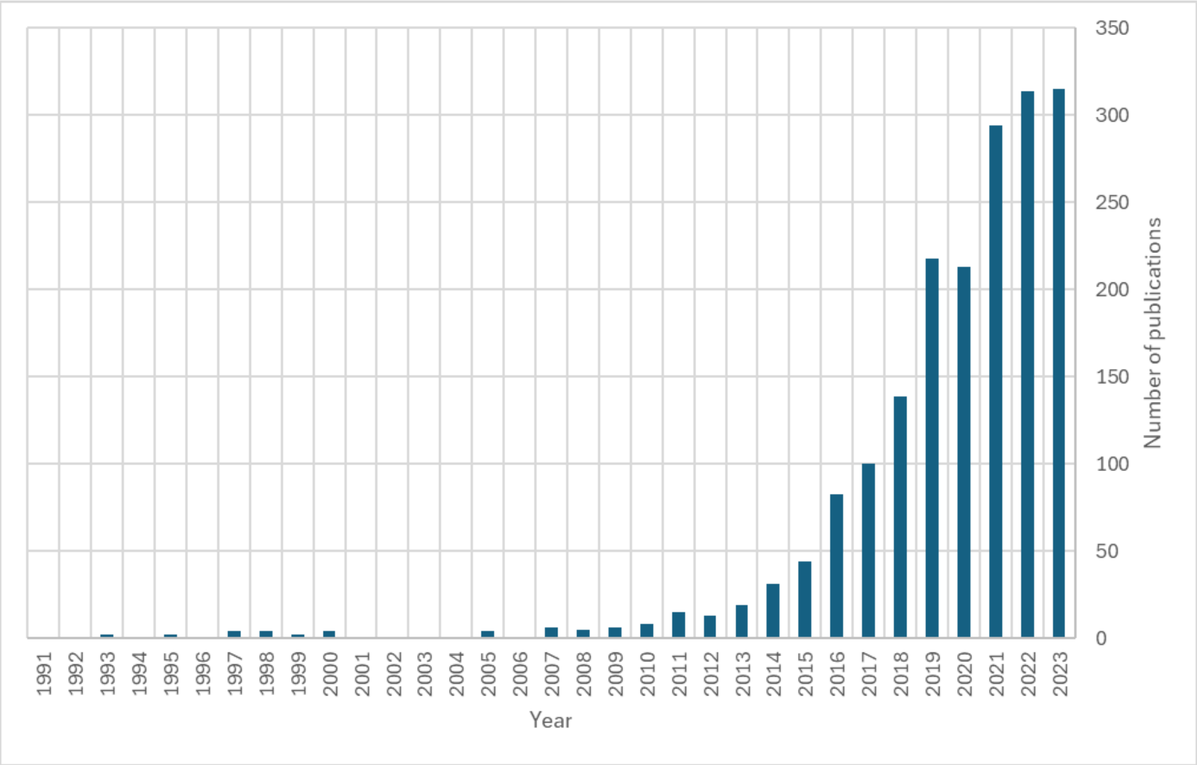

Figure S2: Percentage of compounds containing heteroatoms which can be selectively detected by ICP-MS (from the NORMAN list of Emerging contaminants current status 106).

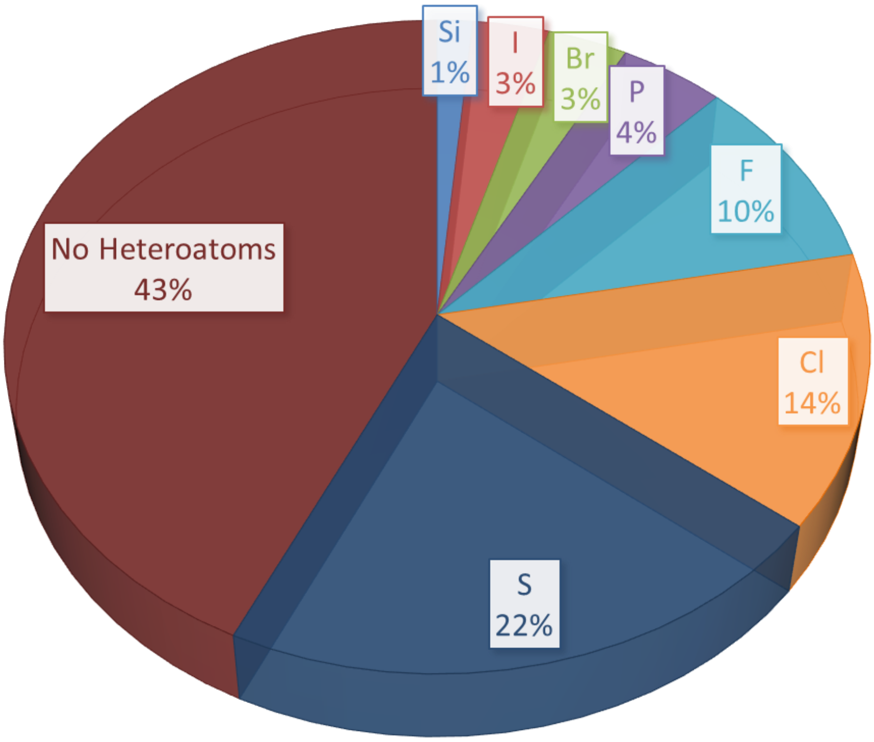

Figure S3: Identification Level scheme modified from Schymanski et al. 2014 by introducing ICP-MS information to the scheme as level 4b. Its element-specific parallel detection enhances the confidence for the unequivocal molecular formulae.

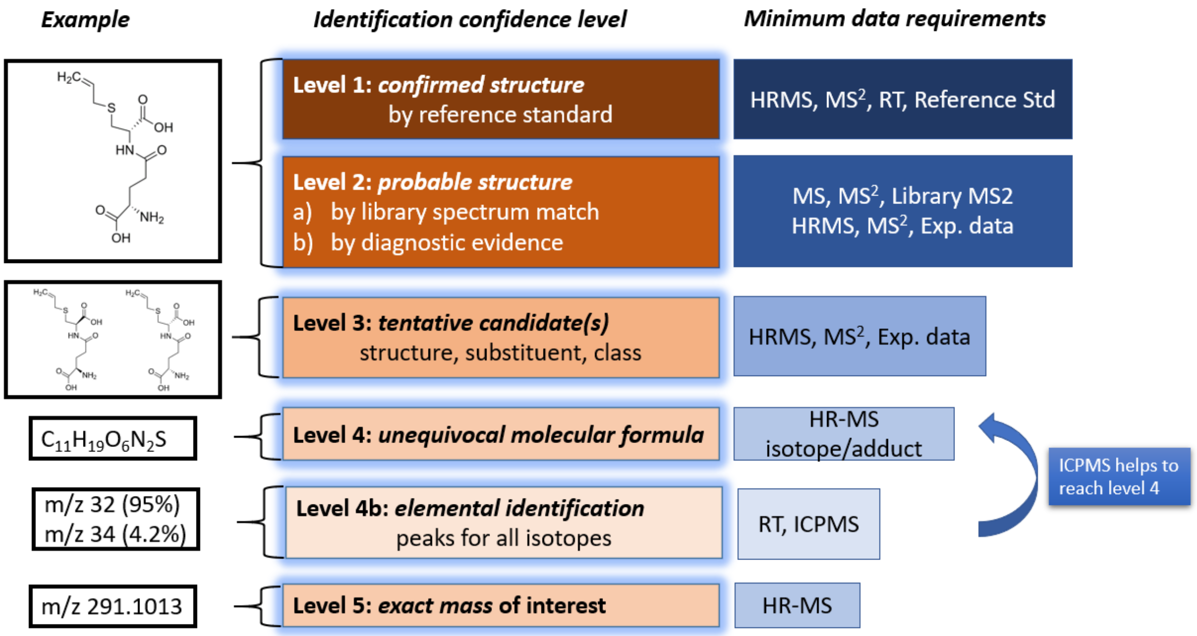

Figure S4: Confidence level for quantification using NTS. We suggest six levels of confidence in quantitative data. Level 6 uses a surrogate standard and has the lowest level of confidence, while level 5 uses an identical standard for external calibration. Using ICP-MS can increase the confidence because of its element-specific detection with low matrix effect of compounds that contain a hetero-atom detectable with ICP-MS. Level2 and 1 are using isotopically-labelled standards spiked at different times to the sample workflow.

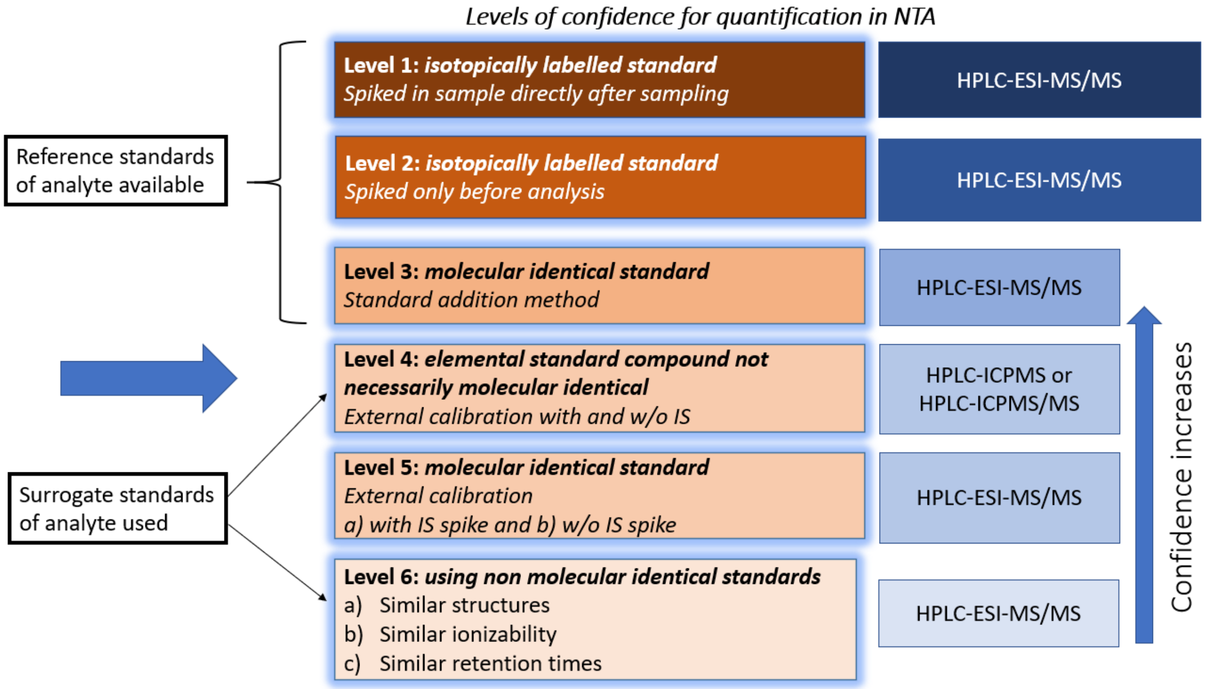

Supplement: Supplementary file 1 — es4c00504_si_001.pdf [file es4c00504_si_001.pdf]
